# Unveiling the Incidence and Graft Survival Rate in Kidney Transplant Recipients With *De Novo* Thrombotic Microangiopathy: A Systematic Review and Meta-Analysis

**DOI:** 10.3389/ti.2024.12168

**Published:** 2024-01-23

**Authors:** Chien-Ya Hsiung, Hsin-Yu Chen, Shih-Han Wang, Ching-Ying Huang

**Affiliations:** ^1^ Division of Gastroenterology, Department of Internal Medicine, Kaohsiung Veterans General Hospital, Kaohsiung, Taiwan; ^2^ Fu Jen Catholic University, New Taipei City, Taiwan; ^3^ Division of Nephrology, Department of Internal Medicine, Kaohsiung Veterans General Hospital, Kaohsiung, Taiwan; ^4^ School of Medicine, National Yang-Ming University, Taipei, Taiwan; ^5^ Department of Pharmacy, Kaohsiung Medical University Hospital, Kaohsiung Medical University, Kaohsiung, Taiwan; ^6^ Department of Pharmacy, Kaohsiung Veteran General Hospital, Kaohsiung, Taiwan

**Keywords:** thrombotic microangiopathy, kidney allograft, renal function, graft survival rate, graft loss rate

## Abstract

*De novo* thrombotic microangiopathy (TMA) is a rare and challenging condition in kidney transplant recipients, with limited research on its incidence and impact on graft survival. This study conducted a systematic review and meta-analysis of 28 cohorts/single-arm studies and 46 case series/reports from database inception to June 2022. In meta-analysis, among 14,410 kidney allograft recipients, *de novo* TMA occurred in 3.20% [95% confidence interval (CI): 1.93–4.77], with systemic and renal-limited TMA rates of 1.38% (95% CI: 06.5–2.39) and 2.80% (95% CI: 1.27–4.91), respectively. The overall graft loss rate of *de novo* TMA was 33.79% (95% CI: 26.14–41.88) in meta-analysis. This study provides valuable insights into the incidence and graft outcomes of *de novo* TMA in kidney transplant recipients.

## Introduction

Thrombotic microangiopathy (TMA) is a rare complication of kidney transplantation that is often associated with poor graft and patient outcomes. TMA can be diagnosed based on clinical or histopathological features. Clinical recognition of TMA requires evidence of (a) microangiopathic hemolytic anemia: fragmented red blood cells on a peripheral blood smear, decreased haptoglobin levels, elevated lactate dehydrogenase and indirect bilirubin levels, and a decline in hemoglobin levels; (b) thrombocytopenia; and (c) evidence of organ damage. The common sites are the kidneys, central nervous system, and gastrointestinal tract [1]. Allograft biopsy is the gold standard method for establishing the diagnosis. Histologically, TMA is characterized by the patchy distribution of the vessel wall and detachment of edematous endothelial cells from the basement membrane. This causes intravascular platelet aggregation with subsequent formation of platelet-rich thrombi within the microcirculation and obstruction of vessel lumina [2].

Post-transplant TMA is classified into recurrent TMA and *de novo* TMA. Recurrent TMA is characterized by the same disease process that manifests as TMA involving the native kidney and recurs in the case of the allograft. In contrast, *de novo* TMA develops for the first time in kidney transplant recipients who had no evidence of the disease before transplantation. A study based on the United States Renal Data System indicated that the incidence of overall TMA in kidney allograft recipients was 5.6 episodes per 1,000 person-years, with approximately 50% patient mortality at 3 years [3]. As for *de novo* TMA, the incidence had been reported with a wide range and could be incorrectly estimated due to missed diagnosis of TMA before kidney transplantation. The incidence of *de novo* TMA has been reported to range from 3% to 14%, and the allograft loss rate ranges from 10% to 57%, both with a wide range [4]. *De novo* TMA not only causes acute decline of allograft function but also different degrees of sequelae. Graft loss in the case of *de novo* TMA is up to 40% within 2 years of diagnosis [3]. Outcomes range from transient renal dysfunction with mild clinical significance to acute renal failure requiring temporary dialysis therapy, potential allograft loss, and patient mortality. The outcome depends on the histopathological severity of the TMA, the promptness of the diagnosis, and the initiation of treatment [5].

The etiologies of kidney allograft *de novo* TMA include calcineurin inhibitors (CNIs), mammalian target of rapamycin inhibitors, ischemia-reperfusion injury, antibody-mediated rejection (AMR), viral infection, thrombotic thrombocytopenic purpura, and atypical hemolytic uremic syndrome (aHUS). CNIs, both cyclosporine and tacrolimus, are well-documented medications that cause *de novo* TMA [6–11]. Mammalian target of rapamycin inhibitors, such as sirolimus and everolimus, comprise much of the drug-related etiologies of TMA [12–15]. AMR is also a common and well-recognized cause of post-transplant TMA [16, 17]. Other less common causes, which can lead to TMA, include various viral infections such as infection of hepatitis C, cytomegalovirus, parvovirus, and BK virus [18–23]. Antiviral therapy [24], disseminated histoplasmosis [25], and thrombotic thrombocytopenic purpura are also among the reported etiologies [26–28]. aHUS is also an important cause. The presence of genetic mutations in complement systemic regulation can trigger an uncontrolled alternative complement pathway activity, resulting in endothelial injury, the pathogenetic basis of TMA [29].

Existing evidence about the incidence and outcome of *de novo* TMA is mainly based on case series and retrospective studies, comprising a wide range of data. Studies on the incidence and graft outcomes of *de novo* TMA are lacking. Therefore, this study aimed to present comprehensive data on the incidence, graft loss, and survival of kidney allografts in patients with *de novo* TMA.

## Materials and Methods

### Study Design

We conducted a systematic review and meta-analysis to evaluate the incidence and survival of kidney allografts in patients with *de novo* TMA. This systematic review was conducted in accordance with the Preferred Reporting Items for Systematic Reviews and Meta-analyses guidelines and The Cochrane Collaboration form [30, 31].

### Search Strategy and Eligibility Criteria

We systematically searched PubMed, Cochrane Library, and EMBASE (until April 2022). A manual search of the reference lists of relevant studies was performed to complement our search results. Search terms included kidney transplantation, *de novo*, and thrombotic microangiopathy, including all subheadings of the Medical Subject Headings and text searches for articles that were not indexed. No language restrictions were used to reduce funnel plot asymmetry. Automatic e-mail updates were built to periodically acquire new research results from the databases. Full details of the search strategy are presented in Supplementary Table S1. The reference lists of the relevant reports were manually searched to identify any missing relevant research articles or strategies.

### Study Selection

All randomized controlled trials, observational studies, case reports, and case series were included in this systematic review and meta-analysis if they reported the following: 1) kidney allograft recipient; 2) *de novo* TMA; 3) incidence; or 4) graft survival. The exclusion criteria were as follows: 1) studies without retrievable endpoints; 2) studies with recurrent TMA; and 3) studies with posters or editorial comments only. The titles, abstracts, and contents were screened by three authors (C-YHs, S-HW, and C-YHu) to determine whether the studies met the inclusion criteria. The full texts of potentially relevant studies were retrieved and assessed in more detail.

### Data Extraction

Three reviewers (C-YHu, S-HW, and C-YHs) independently assessed the studies for eligibility and extracted the data using a standardized data extraction form. Disagreements were resolved through a discussion with a fourth author (H-YC). The following parameters were extracted from each study: general characteristics (first author, year of publication, study terms, study design, and country), patient characteristics (number of patients in each treatment arm, patient age, sex, kidney donor types, genetic variants for complement dysregulation, cause of end-stage renal disease [ESRD], anti-rejection regimen, kidney pathological features, treatment of TMA, and follow-up duration), TMA incidence, and kidney allograft survival.

### Quality Assessment

All cohort studies that met the inclusion criteria were subjected to quality appraisal using the Newcastle–Ottawa Scale, which contains 8 items within 3 domains and a total maximum score of 9 for cohort studies. Scores of 7–9 indicate high quality, 4–6 indicate high risk, and 0–3 indicate a very high risk of bias. All the case reports that met the inclusion criteria were subjected to quality appraisal using the CARE checklist and were recorded as “YES,” “PARTLY,” or “NO,” according to information reported by the included studies. The responses were assigned scores of 1, 0.5, and 0, respectively. The overall score was the sum of the 21 sub-items and was defined as “high” (more than 15), “medium” (10.5–14.5), and “low” (less than 10) [32]. These quality assessments were judged independently by two reviewers (S-HW and C-YHs), and any conflict was discussed with the third reviewer (C-YHu).

### Outcomes

The study outcomes were the *de novo* TMA incidence and graft survival rates. *De novo* TMA incidence was divided into systemic and renal-limited TMA. Some studies that were not classified as systemic or renal-limited TMA were classified as unknown type of TMA. Therefore, we reported the following four different TMA incidences: 1) systemic TMA, 2) renal-limited TMA, 3) total TMA, and 4) unknown type of TMA.

The graft outcomes included graft loss and graft survival. Some studies showed graft survival of 1, 2, 3, 4, 5, 8, and 10 years.

### Measurements

*De novo* TMA incidence was reported as a percentage and event per person-year. The pooled estimated incidence of *de novo* TMA was reported with a 95% confidence interval (CI). The graft survival rate was reported as a percentage. The pooled estimated graft survival was also reported with a 95% CI.

### Meta-Analyses

The effect of baseline characteristics on the incidence of *de novo* TMA was analyzed. These factors included C4d, acute AMR, acute cell-mediated rejection, and the use of tacrolimus or cyclosporine. A random effects model was used for the meta-analysis.

### Statistical Analysis

We used the MedCalc statistical software version Medal 20.110 (Acacialaan 22 8400 Ostend Belgium) to conduct meta-analyses and SPSS version 23.0 (IBM Corp., Armonk, New York) for descriptive analyses. Statistical heterogeneity of studies was assessed using I^2^ (inconsistency) from the fixed-effects model. All results were analyzed using a random-effects model if I^2^ was greater than 50% to minimize the potential heterogeneity effect and between-study variance. For descriptive analyses, continuous data were reported as mean ± standard deviation. *p* < 0.05 was considered statistically significant.

## Results

### Study Selection

Overall, 229 potentially relevant articles were identified in the literature search. Based on the review of the titles and abstracts, 126 studies were excluded. Further, 103 full-text articles were assessed for their eligibility; 31 records were excluded for the reasons of posters, insufficient data, or an editorial protocol. Finally, 75 studies met the inclusion criteria. Supplementary Figure S1 summarizes the flowchart of the search. Of the 75 included studies, 46 were case reports and case series, 21 were single-arm studies, and 8 were cohort studies. Eculizumab was approved for aHUS treatment by the Food and Drug Administration of the United States in 2011. Most of the studies published in 2012 collected data before 2012. Therefore, we categorized studies as published before 2013 and after 2013 (Supplementary Figure S2).

### Study Characteristics of Single-Arm and Cohort Studies

Supplementary Table S4 summarizes the characteristics of single-arm and cohort studies. The percentage of males in the included studies ranged from 17% to 77.8%. The recruitment years of the studies ranged from 1980 to 2019, and 13 studies were published before 2013. The mean age was not reported in 9 studies, while the mean age reported in the other 20 studies was >23 years. The proportion of sex was not reported in 15 studies, whereas the male sex percentage was ranged from 0% to 77.7% in 14 studies. The study population was divided into kidney allograft recipients and renal biopsy recipients. The causes of ESRD included presumed chronic glomerulonephritis, presumed chronic interstitial nephritis, IgA nephropathy, focal segmental glomerulosclerosis, diabetic nephropathy, nephrosclerosis, lupus nephropathy, polycystic kidney disease, and hypertensive nephrosclerosis; however, they were not reported in 22 studies. Regarding management, CNI adjustment was reported in seven studies, two studies reported the efficacy of plasma exchange (PE), and three studies reported eculizumab therapy. Moreover, the proportion of AMR was mentioned in five studies, whereas the proportion of ABO-incompatible cases was mentioned in one study. Finally, one study reported pregnancy outcomes.

### Study Characteristics of Case Reports and Case Series

Supplementary Table S2 and Supplementary Figure S3 summarize the characteristics of the case reports and case series. A total of 46 case reports and case series of 62 kidney allograft *de novo* TMA recipients were identified. A total of 42 (68%) recipients were tacrolimus users, 15 (24%) were cyclosporine users, and 5 (8%) were sirolimus users. The gene mutation data were limited. Of the 42 tacrolimus users, 9 possessed a complement factor H mutation, 2 possessed a complement factor I mutation, 1 possessed a factor II mutation, and 1 had a factor V mutation. Further, 5 of the 62 patients had a history of kidney transplants, 20 were living donor recipients, and 38 were deceased donor recipients. The onset timings (mean ± SD) of TMA were 11.26 ± 37.38, 16.68 ± 32.99, and 1.71 ± 2.96 months among tacrolimus, cyclosporine, and sirolimus users, respectively.

Six patients had AMR, six had cell-mediated rejection, and six had C4d+ on kidney pathology. Five patients were ABO-incompatible. The management of TMA included tapering the CNI and sirolimus dose, and then shifting to other immunosuppressive agents, eculizumab therapy, PE or infusion therapy, or belatacept therapy. A total of 34 (55%) patients received PE or infusion, and 18 (29%) patients received eculizumab therapy. The follow-up periods, months (mean ± SD) were 19.10 ± 37.23, 14.81 ± 13.74, and 4.45 ± 4.68 among tacrolimus, cyclosporine, and sirolimus users, respectively. Finally, 8 of the 62 individuals showed graft loss, whereas 48 individuals showed improvement in serum creatinine levels.

### Incidence of *De Novo* Thrombotic Microangiopathy

The detailed *de novo* TMA incidence in the individual studies is summarized in Table 1. Among the studies included in our analysis, 20 reported on the incidence of *de novo* TMA. Of them, 18 studies focused on *de novo* TMA in kidney allograft recipients, whereas the remaining 2 studies [16, 36] reported on *de novo* TMA detected in kidney allograft biopsies. Two studies reported only on the incidence of TMA, without specifying the number of kidney allograft recipients or biopsies involved [34, 36]. Therefore, these studies were excluded from the meta-analysis.

**TABLE 1:** Thrombotic microangiopathy incidence in included cohort and single-arm studies.

Study	Study design	Study population	Total TMA incidence	Systemic TMA incidence	Renal-limited TMA incidence	Study follow-up time (month)	C4d+	C4d−	Tacrolimus base regimen	Cyclosporine base regimen	Acute antibody-mediated rejection	Acute cell-mediated rejection	ABO incompatible
Baid, 1999 [33]	Single-arm	KAR	3.2% (12/379)		3.2% (12/379)	84							
Braet, 2016 [34]	Single-arm	KAR	2.20%		N/A	428							
Caires, 2012 [35]	Single-arm	KAR	1.1% (17/1,549)		1.1% (17/1,549)	132							
Dessaix, 2019 [36]	Single-arm	KAB	4.80%			6.6							
Doradla, 2020 [37]	Single-arm	KAR	0.85% (17/2,000)	0.2% (4/2,000)	0.65% (13/2,000)								
Fortin, 2004 [38]	Cohort	KAR	3.53% (13/368)		3.53% (13/368)				1.29% (3/233)	3.70% (2/54)			
Franco, 2003 [39]	Single-arm	KAR	0.26% (10/3,862)	0.26% (10/3,862)		24							
Futamura, 2020 [40]	Single-arm	KAR	5.16% (69/1,336)			211							
Gumber, 2014 [41]	Single-arm	KAR	2.89% (34/1,175)			72							
Kocak, 2015 [42]	Single-arm	KAR	2.72% (13/477)	2.72% (13/477)		36			2.72% (13/477)				
Langer, 2001 [43]	Single-arm	KAR	1.5% (10/672)	1.5% (10/672)		212				1.5% (10/672)			
Nava, 2014 [44]	Cohort	KAR	7.3% (36/496)			180				N/A			
Oyen, 2006 [45]	Single-arm	KAR	0.82% (7/850)			48				0.82% (7/850)			
Ozedemir, 2018 [46]	Single-arm	KAR									33.33% (30/90)	17.6% (9/51)	
Reynolds, 2003 [3]	Cohort	KAR	4.9/1,000 PY										
Santos, 2003 [47]	Single-arm	KAR	5% (6/115)	5% (6/115)									
Satoskar, 2010 [16]	Single-arm	KAB					13.6% (33/243)	23.6% (6/715)					
Schwimmer, 2003 [4]	Single-arm	KAR	3% (21/742)	1.07% (8/742)	1.75% (13/742)				52% (11/21)	48% (10/21)			
Tasaki, 2019 [17]	Cohort	KAR	7.5% (15/201)	7.5% (15/201)		214							17.2% (15/87)
Zarifian, 1999 [48]	Single-arm	KAR	13.8% (26/188)	1.06% (2/188)	12.7% (24/188)								

KAR, kidney allograft recipients; KAB, kidney allograft biopsies; PY, person-years; TMA, thrombotic microangiopathy.

**FIGURE 1:**
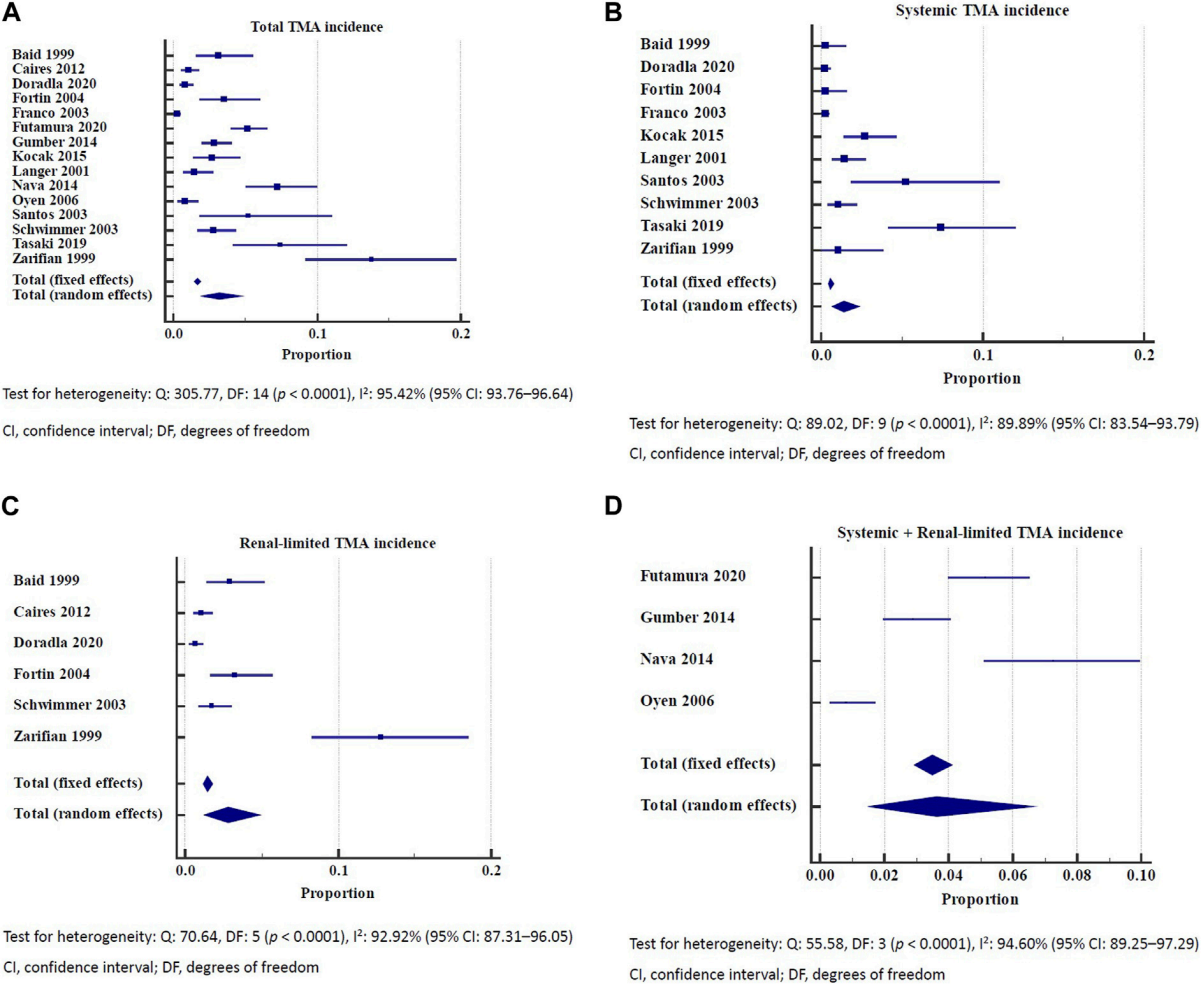
**(A)** Incidence of total *de novo* thrombotic microangiopathy. Test for heterogeneity: Q: 305.77, DF: 14 (*p* < 0.0001), I^2^: 95.42% (95% CI: 93.76–96.64). CI, confidence interval; DF, degrees of freedom. **(B)** Incidence of systemic thrombotic microangiopathy. Test for heterogeneity: Q: 89.02, DF: 9 (*p* < 0.0001), I^2^: 89.89% (95% CI: 83.54–93.79). CI, confidence interval; DF, degrees of freedom. **(C)** Incidence of renal-limited thrombotic microangiopathy. Test for heterogeneity: Q: 70.64, DF: 5 (*p* < 0.0001), I^2^: 92.92% (95% CI: 87.31–96.05). CI, confidence interval; DF, degrees of freedom. **(D)** Incidence of systemic and renal-limited thrombotic microangiopathy. Test for heterogeneity: Q: 55.58, DF: 3 (*p* < 0.0001), I^2^: 94.60% (95% CI: 89.25–97.29). CI, confidence interval; DF, degrees of freedom.

Among kidney allograft recipients, the overall incidence of *de novo* TMA was 3.2% (95% CI: 1.93–4.77) (Figure 1A). The incidence of systemic and renal-limited *de novo* TMA was 1.38% (95% CI: 0.65–2.39) and 2.79% (95% CI: 1.27–4.91), respectively (Figures 1B, C). The unknown type of TMA incidence was 3.64% (95% CI: 1.50–6.67) (Figure 1D). All the outcomes showed significant heterogeneity (I^2^ > 89%). Stratifying the analysis based on distinct follow-up periods provided information on the overall incidence of thrombotic microangiopathy (TMA). Within 5 years follow up time, the TMA incidence was 1.04% (95% CI: 0.16–2.68) with significant heterogeneity (I^2^: 92.17%) (Supplementary Figure S4). As the follow-up duration extended to the 5–10 years, the TMA incidence was 3.02% (95% CI: 2.23–3.92) with low heterogeneity (I^2^: 0.00%) (Supplementary Figure S5). If follow-up was more than 10 years, the TMA incidence was 4.15% (95% CI: 1.64–7.75) with significant heterogeneity (I^2^: 95.54%) (Supplementary Figure S6). In a cohort study conducted in 2003, the incidence of *de novo* TMA in kidney allograft recipients was 4.9 episodes per 1,000 person-years [3].

The incidence of *de novo* TMA among kidney allograft biopsies ranged from 0.26% to 4.8% across the studies [34, 36, 39]. The incidence of systemic *de novo* TMA was 0.26% [39].

### Graft Survival Rate in Patients With *De Novo* Thrombotic Microangiopathy

**FIGURE 2:**
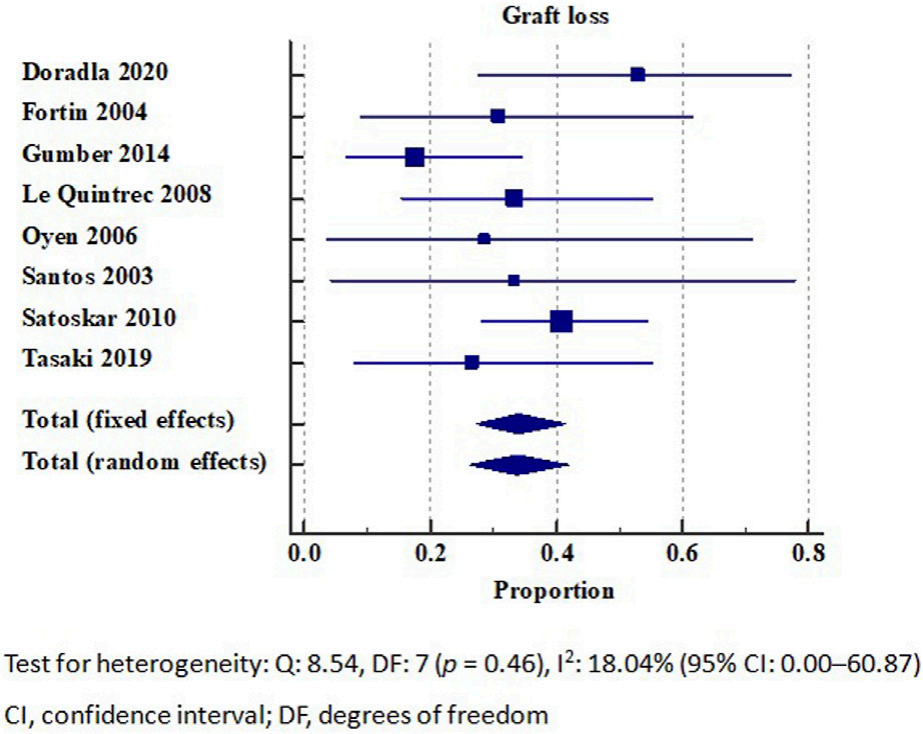
Graft loss in patients with *de novo* thrombotic microangiopathy. Test for heterogeneity: Q: 8.54, DF: 7 (*p* = 0.46), I^2^: 18.04% (95% CI: 0.00–60.87). CI, confidence interval; DF, degrees of freedom.

The detailed individual *de novo* TMA graft survival rate is summarized in Table 2. Our analysis included a total of 18 studies that reported on kidney allograft survival, of which, 8 were eligible for inclusion in the meta-analysis [16, 17, 37, 38, 41, 45, 47, 50]. The overall graft loss rate of *de novo* TMA was 33.79% (95% CI: 26.14–41.88). No significant heterogeneity (I^2^ = 18.04%) was observed (Figure 2). The meta-analysis of seven studies reporting 1-year graft survival outcomes revealed a rate of 55.39% (95% CI: 36.46–73.54). However, a substantial degree of heterogeneity (I^2^ = 88.12%) was observed (Supplementary Figure S7).

**TABLE 2:** Graft outcome in included cohort and single-arm studies.

		Graft loss							Graft survival						
Study	Study design	Total graft loss	Plasma exchange	Either tacrolimus or cyclosporine, shift to sirolimus (%)	Acute antibody-mediated rejection (%)	Acute cell-mediated rejection (%)	C4d+	C4d− (%)	1-year survival (%)	2-year survival (%)	3-year survival (%)	4-year survival (%)	5-year survival (%)	8-year survival (%)	10-year survival (%)
Baid, 1999 [33]	Single-arm								40						
Braet, 2016 [34]	Single-arm								32						
Caires, 2012 [35]	Single-arm											43			
Costa, 2013 [49]	Single-arm								73.30						
Dessaix, 2019 [36]	Single-arm								8						
Doradla, 2020 [37]	Single-arm	53% (9/17)			100	100			47				35		35
Fortin, 2004 [38]	Single-arm	30.77% (4/13)													
Gumber, 2014 [41]	Single-arm	17.65% (6/34)													
Le Quintrec, 2008 [50]	Single-arm	33.33% (8/24)							67						
Meehan, 2011 [51]	Single-arm						57%								
Oyen, 2006 [45]	Single-arm	28.57% (2/7)		28.6											
Ozedemir, 2018 [46]	Single-arm								83	51			51		
Reynolds, 2003 [3]	Cohort								47	35					
Santos, 2003 [47]	Single-arm	33.33% (2/6)													
Satoskar, 2010 [16]	Single-arm	40.68% (24/59)	35% (8/23)				40%	42							
Tasaki, 2019 [17]	Cohort	26.67% (4/15)													
Wu, 2016 [52]	Cohort								70.0	48.3				28.0	
Zarifian, 1999 [48]	Single-arm								81		69				

33% of patients with CNI-related TMA (4 out of 12) developed ESRD, while all patients with rejection-associated TMA developed ESRD [37]. The overall 1-year graft survival rate was 47%, whereas the 5- and 10-year graft survival rates were 35%. Additionally, there was no significant difference in the graft survival rate between the renal-limited and systemic TMAs (*p* = 0.4) [37].

In one study, among 33 C4d-positive TMA patients, 23 (70%) underwent plasmapheresis, with a graft loss rate of 35% (8 out of 23). Conversely, the remaining 30% (10 patients) did not receive plasmapheresis, and among these, the graft loss rate was higher at 50% [16].

### Study Quality of Included Cohort Studies

All observational studies scored from 6 to 9 on the Newcastle–Ottawa Scale criteria and were included in the quantitative analysis (Supplementary Table S3). Five cohort studies were considered to be of high quality (Newcastle–Ottawa score ≥ 7).

## Discussion

Our systematic review and meta-analysis encompassed 75 studies, including 29 cohort or single-arm studies and 46 case series or case reports, to provide a comprehensive examination of the incidence and graft survival rate in kidney allograft recipients with *de novo* TMA. Among 14,410 kidney allograft recipients, 306 individuals developed *de novo* TMA, corresponding to an incidence of 3.20%, while the incidences of systemic TMA and renal-limited TMA were 1.38% and 2.80%, respectively. Among the 200 kidney allograft recipients who developed *de novo* TMA, 138 individuals remained dialysis-free 1 year after transplantation. However, among the 175 individuals with *de novo* TMA who were followed up for graft outcomes, 59 individuals eventually experienced graft loss, resulting in an overall graft loss rate of 33.79%.

Data on the incidence difference between kidney recipients with renal-limited TMA and systemic TMA are inconsistent. A study involving 21 individuals with pathology-proven kidney allograft TMA showed that 60% of the individuals had systemic TMA, and 40% had renal-limited TMA [4]. However, in contrast, only 5% of 43 individuals with pathology-proven lupus nephritis and concomitant TMA were found to have systemic TMA [53]. Moreover, comparative studies investigating differences in graft survival rates between patients with renal-limited and systemic TMA are scarce. In a case series involving 21 individuals with kidney allograft TMA, including 8 with renal-limited TMA and 13 with systemic TMA, Kaplan–Meier analysis revealed that those with renal-limited TMA had better graft survival than those with systemic TMA, with an average follow-up of 62 months [4]. Therefore, a well-designed future study is needed to examine the difference in incidence and outcomes between renal-limited and systemic TMA.

More than 90% of kidney recipients are treated with CNIs, and only a few develop *de novo* TMA. Therefore, caution should be exercised before attributing *de novo* TMA to CNIs until other predisposing factors have been ruled out [54]. The incidence of CNIs-related *de novo* TMA ranges from 1.29% to 3.7% [43, 55]. Several mechanisms explain the relationship between CNIs and *de novo* TMA. In CNI users, an imbalance of vasodilators (prostaglandin E2, prostacyclin I2, and nitric oxide) and vasoconstrictors (thromboxane A2 and endothelin) leads to glomerular arteriolar vasoconstriction and endothelial damage [56, 57]. The release of microparticles from CNIs-exposed endothelium is reported to activate the complement alternative pathway, causing endothelial cell damage [11]. Our analysis of case reports and series revealed that tapering down the CNIs-dose is the most common strategy, followed by shifting to other CNIs or sirolimus. However, the graft survival rate remains unfavorable and is reported to be 28.6% [45].

C4d is an indicator of an activated classical complement pathway, and linear C4d staining in the peritubular capillary is a key diagnostic feature of AMR [58]. A retrospective study involving 59 individuals with kidney allograft TMA revealed that those with peritubular capillaries linear C4d staining had a nearly 4-fold higher incidence of TMA than did those without C4d staining (C4d+ vs. C4d−: 13.6% vs. 3.6%) [16]. However, the 2-year graft loss rate was similar between the two groups, with nearly 40% in each group. In contrast, another study of 74 individuals with kidney allograft TMA found that those with C4d+ had a higher graft loss rate than those without C4d staining (55.6% vs. 30%) [46]. Nevertheless, C4d deposits are not uncommon in kidney allograft TMA, particularly in the glomeruli. In a study of 32 individuals with renal TMA, which included 12 kidney allograft sections and 30 native kidney sections, C4d deposits were detected in 88% of TMA cases, while C5b-9 deposits were detected in 76% of TMA cases [58]. Notably, of the 12 kidney allograft TMA sections, C4d deposits were present in 75% of glomeruli, and C5b-9 deposits were present in 50%. The study showed that C4d and C5b-9 are common denominators in kidney allografts in patients with TMA and suggested that anti-terminal complement therapy may be beneficial in these patients. The management strategy for *de novo* TMA includes identifying and removing triggers, PE, and eculizumab therapy. However, the efficacy of PE in *de novo* TMA has not been fully established, owing to the heterogeneity of its etiologies. Although PE plays an important role in managing thrombotic thrombocytopenic purpura, it is also used as a bridging therapy to eculizumab in patients with aHUS. In a single-arm retrospective cohort study conducted in the United States in 2003 (pre-eculizumab era) to examine the efficacy of PE in 29 kidney allograft recipients with TMA, 6 (20%) of them suffered from graft loss. Among the 10 individuals who had histological acute rejection, 6 (60%) suffered from graft loss within 1 year [59]. In a comparative study conducted in 2010 (pre-eculizumab era), which aimed to explore the efficacy of PE with concurrent intravenous immunoglobulin in 33 kidney allograft recipients with *de novo* TMA and concomitant AMR, the graft loss rate was not different between those with and without PE + intravenous immunoglobulin (35% vs. 50%) [16]. In a single-arm retrospective cohort study conducted in Spain in 2020, which comprised 16 kidney allograft recipients with *de novo* aHUS, only 2 of 13 individuals who underwent PE achieved complete hematological and renal recovery. Eight individuals received rescue eculizumab owing to no or partial renal response to PE, and six (75%) of them achieved complete hematological and renal recovery after receiving rescue eculizumab [60]. Finally, according to our analysis of case reports and series, 52% of 48 individuals with *de novo* TMA who underwent PE achieved a renal response, whereas 83% of 18 individuals with *de novo* TMA receiving eculizumab achieved a renal response.

This study has few limitations. First, owing to the low prevalence of *de novo* TMA, there was a lack of randomized controlled trials, and the meta-analysis results were based on single-arm or observational cohort studies. Additionally, there was a wide variance in the number of cases among the enrolled studies. Second, there was heterogeneity in our meta-analysis of TMA incidence, which may be mainly attributed to the influence of the following three studies: a study by Tasaki et al., which included ABO-incompatible kidney allograft recipients and had a high TMA incidence; a study by Nava et al., which included older kidney allograft recipients and had a high TMA incidence; and a study by Zarifian et al., which had a higher proportion of individuals with chronic transplant nephropathy and was conducted in 1999 when all recipients were receiving cyclosporine for immunosuppression [17, 44, 53]. Finally, 9 of the 15 studies included in our TMA incidence meta-analysis and 5 of 8 studies included in our graft survival meta-analysis were conducted before 2013 (pre-eculizumab era). This may have led to a bias in both TMA incidence and graft survival rates in the current eculizumab era.

To conclude, the incidence of *de novo* TMA in patients with kidney allografts was 3.20%, whereas the incidences of systemic TMA and renal-limited TMA were 1.38% and 2.80%, respectively. The overall graft loss rate was 33.79%. These findings highlight the rare and complex nature of *de novo* TMA in kidney allograft recipients, which is associated with poor graft outcomes. Our study provides valuable insights into the incidence and graft outcomes.

## Abbreviations

aHUS, atypical hemolytic uremic syndrome; AMR, antibody-mediated rejection; CI, confidence interval; CNIs, calcineurin inhibitors; ESRD, end-stage renal disease; PE, plasma exchange; TMA, thrombotic microangiopathy.
