# Supplementary material for: Unveiling the Incidence and Graft Survival Rate in Kidney Transplant Recipients With De Novo Thrombotic Microangiopathy: A Systematic Review and Meta-Analysis
Source: Transpl Int. 2024 Jan 23;37:12168. doi: 10.3389/ti.2024.12168 (PMC10844394; doi:10.3389/ti.2024.12168)

**Supplementary contents**

Capsule Sentence Summary

Table S1. Search strategies

Table S2. Characteristics of patients with de novo thrombotic microangiopathy caused by different immunosuppressants according to case series and reports

Table S3. Study quality of enrolled cohort studies

Table S4. Characteristics of the included cohort and single-arm studies

Figure S1. PRISMA flow chart summarizing study identification and selection

Figure S2. A matrix indicating enrolled study type versus published year

Figure S3. Matrix indicating immunosuppressants versus demographics, treatments, and outcomes according to case series and reports

Figure S4. Incidence of de novo thrombotic microangiopathy within 5 years

Figure S5. Incidence of de novo thrombotic microangiopathy between 5 and 10 years

Figure S6. Incidence of de novo thrombotic microangiopathy more than 10 years

Figure S7. One year graft survival in patients with de novo thrombotic microangiopathy

**Capsule Sentence Summary:**

There are limited researches regarding incidence and kidney graft survival of de novo thrombotic microangiopathy (TMA). This meta-analysis of 14,410 kidney allograft recipients reports 3.20% incidence and 33.79% graft loss rate for de novo TMA, highlighting its rarity and impact**.**

**Table S1. Search strategies**

| Database | Key words |
| --- | --- |
| PubMed | ("kidney transplantation"[MeSH Terms] OR ("kidney"[All Fields] AND "transplantation"[All Fields]) OR "kidney transplantation"[All Fields]) AND "de novo"[All Fields] AND ("thrombotic microangiopathies"[MeSH Terms] OR ("thrombotic"[All Fields] AND "microangiopathies"[All Fields]) OR "thrombotic microangiopathies"[All Fields] OR ("thrombotic"[All Fields] AND "microangiopathy"[All Fields]) OR "thrombotic microangiopathy"[All Fields]) |
| Cochrane  library | "kidney transplantation" in All Text AND "thrombotic microangiopathy" in All Text - (Word variations have been searched) |
| Embase | ((('de novo' OR (de AND ('novo'/exp OR novo))) AND 'thrombotic microangiopathy'/exp) AND'kidney transplantation'/exp) |

**Table S2. Characteristics of patients with de novo thrombotic microangiopathy caused by different immunosuppressants according to case series and reports**

|  | **Tacrolimus users**  **(n = 42)** | **Cyclosporine users**  **(n = 15)** | | **Sirolimus users**  **(n = 5)** |
| --- | --- | --- | --- | --- |
| **Age, years (mean ± SD)** | 40.95 ± 15.86 | 37.71 ± 13.59 | | 59.86 ± 8.09 |
| **Male sex, n (%)** | 21 (50 %) | 8 (53.33%) | | 3 (60%) |
| **History of previous kidney transplant, n (%)**  **not mentioned** | 4 (9.52 %)  1 | 1 (6.67%)  0 | | 0 |
| **Type of donor, n (%)** |  |  | |  |
| **Living** | 14 | 6 | 0 | |
| **Deceased** | 24 | 9 | 5 | |
| **Not mentioned** | 4 | 0 | 0 | |
| **Gene mutation, n (%)** |  |  |  | |
| **CFH** | 9 | N/A | N/A | |
| **CFI** | 2 | N/A | N/A | |
| **Factor II** | 1 | N/A | N/A | |
| **Factor V** | 1 | N/A | N/A | |
| **No identified mutation** | 6 | 1 | N/A | |
| **Not mentioned** | 23 | 14 | 5 | |
| **Onset timing, months (mean ± SD)**  **not mentioned, n** | 11.26 ± 37.38  2 | 16.68 ± 32.99  0 | 1.71 ± 2.959  0 | |
| **Rejection type** |  |  |  | |
| **Antibody-mediated rejection, n (%)** | 5 | 1 | 0 | |
| **Cell-mediated rejection, n (%)** | 3 | 3 | 0 | |
| **C4d+, n (%)**  **not mentioned** | 6  29 | 0  11 | 0  1 | |
| **CNI toxicity** | 13 | 5 | 0 | |
| **ABO incompatible, n (%)** | 4 | 1 | 0 | |
| **Management** |  |  |  | |
| **CNI based, taper dose** | 31 | 15 | N/A | |
| **Sirolimus based, taper dose** | N/A | N/A | 5 | |
| **Tacrolimus based, shift to sirolimus** | 7 | N/A | N/A | |
| **Cyclosporine based, shift to tacrolimus** | N/A | 3 | N/A | |
| **Cyclosporine based, shift to sirolimus** | N/A | 3 | N/A | |
| **Sirolimus based, shift to cyclosporine** | N/A | N/A | 1 | |
| **Eculizumab** | 18 | 0 | 0 | |
| **Plasma exchange/infusion** | 26 | 4 | 4 | |
| **Belatacept** | 10 | 1 | 1 | |
| **Follow-up periods, months (mean ± SD)** | 19.10 ± 37.23 | 14.81 ± 13.74 | 4.45 ± 4.68 | |
| CFH, complement factor H; CFI, complement factor I; CNI, calcineurin inhibitor; N/A, not applicable; SD, standard deviation | | | | |

**Table S3. Study quality of enrolled cohort studies**

| Newcastle-Ottawa Scale for Assessing the Quality of Cohort Studies | | | | | |
| --- | --- | --- | --- | --- | --- |
| Study, year | Selection | Outcome | Comparability | Total | Study Quality |
| Fortin 2004 | 4 | 3 | 2 | 9 | Good |
| Nava 2014 | 4 | 1 | 1 | 6 | Fair |
| Reynolds 2003 | 4 | 3 | 2 | 9 | Good |
| Schwimmer 2003 | 4 | 3 | 1 | 8 | Good |
| Tasaki 2019 | 4 | 3 | 2 | 9 | Good |
| Wu 2016 | 4 | 3 | 1 | 8 | Good |

**Table S4.** Characteristics of the included cohort and single-arm studies

| **Study** | **Country** | **Study**  **design** | **Age (Mean ± SD)** | **Sex**  **(male %)** | **Underlying disease** | **Recruitment year** | **Intervention** | **Comparison** | **Outcome** |
| --- | --- | --- | --- | --- | --- | --- | --- | --- | --- |
| Baid,  1999 (46) | USA | - Single-arm - Total 379 patients with kidney transplantation | 43.1 ± 9.5 | 61% | N/A | May 1990– December 1996 | 18 recipients (4.8%) who had HCV-positive serology | N/A | - TMA: 5 patients; mean follow-up, 29 ± 29.4 months - No-TMA: 13 patients; mean follow-up, 29.9 ± 27.3 months |
| Braet, 2016 (47) | Belgium | - Retrospective cohort - Total 67 patients with thrombi in pathology or coded diagnosis of TMA | N/A | N/A | N/A | June 1980– August 2015 | - Total 53 patients with de novo TMA - Kidney transplant patients with either thrombi on post-transplant renal allograft biopsy or TMA coded diagnosis were included, then excluded recurrent TMA | N/A | - De novo TMA incidence: 2.2% - Graft failure within 1 year: 32% |
| Caires,  2012 (48) | Brazil | - Single-arm - Total 1,549 patients with kidney transplantation | 40 ± 15 | N/A | N/A | 2000–2010 | - Calcineurin inhibitor withdrawal or reduction was the first step in the management of 10/15 (66%) patients, and 6 (35%) received fresh frozen plasma and/or plasmapheresis - Tacrolimus was successfully reintroduced in 6 patients after a median of 17 days | N/A | - TMA occurred at a median of 25 days (range: 1–1755) after transplantation - 9 (53%) patients developed TMA within 1 month of transplantation; 12% developed TMA after 1 year - 8 (47%) patients needed dialytic support after TMA diagnosis, and 75% remained on dialysis |
| Costa,  2013 (49) | Spain | - Single-arm - Total 627 kidney biopsies | N/A | N/A | N/A | N/A | 627 kidney biopsies   - De novo TMA: 14 (2.2%) | - Transplant glomerulopathy: 39 (6.2%) - Transplant glomerulopathy was diagnosed later than de novo TMA (6.9 ± 5.9 vs. 3.5 ± 6.5 years, *p* = 0.01) and patients presented higher proteinuria (4.0 ± 3.6 vs. 2.3 ± 1.6 g/24 h, *p* = 0.02) | Graft loss rate was similar in both groups (47.1% vs. 45.5%, *p* = 0.93) |
| Dessaix,  2019 (50) | France | - Single-arm - Total 2,042 patients with kidney transplantation | N/A | N/A | N/A | January 2004– March 2016 | - 98 patients experienced at least one episode of histological TMA; 90% had de novo TMA (4.8% of the kidney transplant population) - Median time of occurrence: 198 ± 920 days | Kidney transplant recipients without TMA | - Antibody-mediated rejection (46.9%) - Underlying abnormalities of the alternative complement pathway (10.2%) - The two main factors associated with graft loss were score of intimal arteritis and renal function at the time of diagnosis - Graft survival was worst compared with kidney transplant recipients without TMA (84.7% vs. 91.3% at 5 years; *p* < 0.0001) - 1-year graft loss   - De novo TMA: 8%  - Recurrent TMA in 10% patients: 8% |
| Doradla,  2020 (51) | India | - Retrospective single-arm - Total 2,000 patients with kidney transplantation - 17 patients with TMA | 39.59 ± 11.08 | 70.50% | - Presumed chronic glomerulonephritis: 11/17 (64.7%) - Presumed chronic interstitial nephritis: 3/17 (17.6%) - Biopsy-proven IgA nephropathy: 1/17 (5.8%) - Biopsy-proven focal segmental glomerulosclerosis: 2/17 (11.6%) | 1989–2015 | - 17 patients with TMA out of 2,000 kidney transplant patients   - Cyclosporine: 7/17 (41.1 %)  - Tacrolimus: 10/17 (58.8 %)   - 12 patients diagnosed with CNI-related TMA   - 6 received CNI dose reduction  - 3 switched to mTOR inhibitors  - 3 switched to other CNI | - Rejection-associated TMA (n = 5)  - rescued with ATG + PEX: 4/5 (80%)  - rescued with ATG alone: 1/5 (20%) | - 1-year graft survival rate: 47% - 5- and 10-year graft survival rate: 35% |
| Elharrif,  2019 (52) | USA | - Single-arm - 45 patients with kidney transplantation | 54 ± 11.9 | 45% | N/A | 2013–2019 | - 70% patients with pathology-proven TMA - 45 kidney transplant patients switched from CNIs to belatacept for suspect TMA. - Post switch follow-up of 529 days | N/A | After switch to belatacept:  - Significant improvement in serum creatinine level (4.39 vs. 2.0 ± 1.4 mg/dL, *p* = 0.001)  - Significant improvement in urine protein/creatinine level (*p* = 0.006)  - No significant change in donor specific anti-HLA antibodies titers |
| Fortin,  2004 (53) | Canada | - Cohort - Kidney and   kidney-pancreas  transplant  recipients (n = 368) | - Patients with TMA: 37.9 ± 8.3 - Patients without   TMA: 45.7 ± 12.6 | N/A | - Patients with TMA   -Diabetes mellitus: n = 4  -Hypertension: n = 2  -Glomerulonephritis: n = 2  -HUS: n = 1  -Others: n = 4   - Patients without TMA   -Diabetes mellitus: n = 71  -Hypertension: n = 14  -Glomerulonephritis: n = 131  -HUS: n = 4  -Others: n = 135 | 1996–2002 | Patients with TMA | Patients without  TMA | - Graft loss rate: 30.7% (4/13) - Incidence of de   novo TMA: 3.5% |
| Franco,  2003 (54) | Spain | - Single-arm - Total 10 patients with de novo TMA | 46.6 ± 14.4 | 70% | Etiology of ESRD:  - Diabetic nephropathy (n = 2)  - Nephrosclerosis (n = 1)  - Lupus nephropathy (n = 2)  - Polycystic kidney disease (n = 1)  - Chronic glomerulonephritis (n = 2)  - Interstitial nephritis (n = 2) | 2000–2001 | - 10 renal transplant recipients who developed de novo TMA.   - Follow-up period: 19 ± 4.3 months  - Calcineurin inhibitors were completely removed and immediate treatment with sirolimus was started after diagnosis | N/A | - Onset time of de novo TMA: 69 ± 80 days after transplantation - Signs of hemolysis: 60% |
| Futamura,  2020 (66) | Japan | - single arm - Total 1336 patients - TMA=69 - Non -TMA=1207 - Exlusion=60(incomplete data) | N/A | N/A | N/A | 2000-2018 | - N/A | N/A | - donor specific antibodies (DSA) (OR 3.52)、use of cyclosporine (OR 3.70) - TMA 5.2% (symptomatic TMA: 0.9%, asymptomatic TMA: 4.3%) |
| Gumber,  2014 (55) | India | - Single-arm - Total 34 patients with de novo TMA | 32.9 ± 9.8 | N/A | N/A | 2008–2013 | - De novo TMA was observed in 34 (2.2%) renal biopsies:   - 25 patients had TMA only, and 9 patients had HUS  - 33 patients had drug-induced TMA (20 with tacrolimus, 12 with cyclosporine, and 1 with sirolimus)  - 1 patient had TMA due to CMV | N/A | - Graft dysfunction in 34 (100%) patients   - Temporary dialysis support: 26 patients  - ESRD: 6 patients |
| Kirsanova,  2018 (56) | Russia | - Single-arm - Total 19 patients with de novo TMA | 29 ± 4.35 | 0% | N/A | 2014–2017 | N/A | N/A | - 19 patients in total   - 10 (52.6%) with laboratory signs of TMA during pregnancy  - 4 (21.3%) with HELLP-syndrome   - Pregnancy outcomes were worse in patients with TMA than in those without TMA   - newborns had low Apgar scores, growth, and birth weights |
| Kocak,  2015 (57) | Turkey | - Single-arm - 477 patients with living-kidney transplantation | N/A | N/A | N/A | 2012–2014 | N/A | N/A | - 13 (2.7%) patients developed de novo TMA - 5 (1.04%) patients needed eculizumab treatment |
| Langer,  2001 (36) | USA | - Single-arm - n = 627 of renal transplant recipients | 45.1 ± 10.1 years (range: 29–61 years) in 10 HUS cases | 50% | N/A | 1993–2000 | CyA + SRL + steroid | N/A | - 1.5% (10 of 672) of renal transplant recipients developed HUS - Risk factor: high drug C0 value, acute rejection episodes, original glomerulopathic disease, and infections |
| Le  Quintrec,  2008 (58) | France | - Single-arm - n = 24 with posttransplant de novo TMA | 46 ± 12 | 62.5% | N/A | N/A | N/A | N/A | - 7 mutations (CFI: 4, CFH: 1, both: 2) in 24 de novo TMA - Control: n = 25, no de novo TMA after renal transplantation, and no genetic abnormality - 8/24 patients had lost their graft (33%), including 3/7 with a mutation (42%) and 5/17 without mutations (29%) |
| Mallett,  2015 (59) | Australia | - Single-arm - Evaluated 10 patients who received compassionate access eculizumab | 23.5 | N/A | N/A | 2010–2014 | Eculizumab | N/A | - 10 patients received compassionate access eculizumab   - De novo TMA: 2  - aHUS recurrence after transplantation: 1 |
| Meehan,  2011 (60) | USA | - Single-arm - Consecutive   renal allograft  biopsies  routinely  stained for C4d  deposition  (n = 1,073 with  adequate tissue  and clinical data  from 563  allografts) | - TMA+C4d+: 46 - TMA+C4d-: 45.3 - TMA-C4d+: 36.4 | - TMA+C4d+: 57.14% - TMA+C4d-: 77.78% - TMA-C4d+: 47.62% | - Diabetic nephropathy (n = 8) - Hypertensive nephrosclerosis (n = 11) - Primary TMA (n = 1) - Primary glomerular disease (n = 8) | December 1, 2004–February 1, 2009 | - TMA+C4d+ - TMA+C4d- | TMA-C4d+ | - Graft loss was significantly greater in early C4d+, TMA+ group than in C4d+ controls without TMA (57% vs. 9.5%, *p* = 0.02) - Incidence of de novo TMA: 3.4% |
| Nava,  2014 (45) | Italy | - Cohort - Patients receiving a renal allograft: 496 patients - Renal graft biopsy specimens: 350 patients | N/A | N/A | N/A | 1998–2012 | - Total 496 patients received kidney transplantation   - 350 renal graft biopsy specimens  - mTORi group de novo TMA: 8 of 153; prevalence, 5.3% | Without mTORi group: 9 of 324; prevalence, 2.8% | - Histologic features of TMA: n = 36 (prevalence, 7.3%) - Drug-related TMA: n = 17 (prevalence, 3.4%) - Non drug-related TMA: n = 19 |
| Oyen,  2006 (40) | Norway | - Single-arm - Total 850 patients with kidney transplantations | N/A | N/A | N/A | 2000–2004 | CNI-free immunosuppression by sirolimus, mycophenolate mofetil, and steroids | N/A | - Primary HUS incidence: 0.82% (7/850) - 8 transplants with previous TMA - 15 primary HUS or previous TMA in total, 13 cases with graft survival in the long term - No TMA/HUS recurrences - High rate of acute rejections: 53% (8/15) |
| Ozedemir,  2018 (42) | Turkey | - Single-arm - Total 272 patients with renal transplantation | 42.8 ± 12.7 | N/A | N/A | N/A | - Total 272 patients   - 74 patients (27.2%) had de novo TMA  - 30 (40.5%) patients with acute humoral rejection  - 9 (12.2%) with acute cellular rejection  - 22 (29.7%) with chronic active humoral rejection  - 10 (13.9%) with polyomavirus nephropathy  - 3 (4.1%) with immunoglobulin A nephropathy | N/A | - De novo TMA 1- year graft survival rate: 83%   - 3-year graft survival rate: 51%  - 5-year graft survival rate: 51% |
| Portoles,  2019 (61) | Spain | - Multicenter retrospective study - Total 15 cases with de novo TMA | 47 | N/A | N/A | N/A | - Total 15 cases with de novo TMA   - 66% with immediate graft  function (2–14 days post Tx)  - 12 cases received eculizumab | N/A | - 12 patients received eculizumab   - Complete remission: 7  - Partial remission: 4  - Nephrectomy: 1   - Eculizumab was withdrawn after 2 months in 11 cases   - One recurrence after 1 year without eculizumab |
| Rabant,  2019 (62) | France | Retrospective cohort | N/A | N/A | N/A | January 2004– March 2016 | N/A | N/A | Median time of occurrence of de novo TMA from kidney transplantation: 198 ± 920 days |
| Reynolds,  2003 (3) | USA | Cohort | - De novo TMA: 40.8 ± 14.2 - Recurrent TMA: 33.4 ± 20.9 - De novo TTP: 40.3 ± 12.3 - Without TMA: 46.0 ± 14.7 | De novo TMA: 37 (46.6%)  Recurrent TMA: 8 (61.5%)  De novo TTP: 12 (40%)  Without TMA: 9496 (60.4%) | De novo TMA: SLE, DM  Recurrent TMA:(-)  De novo TTP: SLE, DM  Without TMA: SLE, DM | January 1, 1998–July 31, 2000 | - De novo TMA - Recurrent TMA - De novo TTP | Without TMA | - Incidence of TMA: 4.9/1,000 patient-year - Graft survival rate   - 1-year: 47%   - - 2-year: 35% Independent risk of mortality:  younger recipient age, older donor age, female recipient, and initial use of sirolimus |
| Santos,  2003 (63) | USA | Single-arm | 34-51 | 17% | Type 1 DM (n = 2), glomerulonephritis (n = 1), IgA nephropathy (n = 1), HTN (n = 1), polycystic kidney disease (n = 1) | Recruitment for 1 year | - OKt3 + (medrol + AZA) n = 1 - OKt3, ATG + (medrol + AZA) n = 2 - Okt3, FK506 + (medrol + AZA) n = 2 - CyA, FK506 + (medrol + AZA) n = 1 | N/A | - Total 6 patients; 2 patients with graft loss   - Graft loss rate: 30% |
| Satoskar,  2010 (16) | USA | Retrospective cohort | N/A | N/A | N/A | January 2003– December 2008 | TMA with C4d positive: 33 patients | TMA with C4d negative: 26 patients | - 2-year graft loss rate   - C4d positive: 13/33(13.6%)  - C4d negative: 11/26 (3.6%) |
| Schwimmer,2003 (4) | USA | - Cohort - 742   kidney and kidney-pancreas transplants: 21 TMA cases | 42.0 ± 10.0 | 52% | N/A | 1985–2000 | - Tacrolimus: 11 (52%) - Cyclosporine: 10 (48%) - Local TMA: 8 (38%) - Systemic TMA: 13(62%) | N/A | Incidence of TMA: 3% (21/742) |
| Tasaki,  2019 (17) | Japan | - Cohort - 201 living-donor kidney transplantation | - ABO-incompatible kidney transplant TMA (n = 15): 38.0 (10-63) - Non-TMA (n = 72): 45.0 (12-68) | In ABO-incompatible kidney transplant TMA group (n = 15): 11 (73.3%) In non-TMA group (n = 72): 52 (72.2%) | Cause of ESRD  TMA group  - IgA nephropathy: 3 (20%)  - DM nephropathy: 2 (13.3%)  Non-TMA group  - IgA nephropathy: 14 (19.5%)  - DM nephropathy: 8 (11.1%)  - PCKD: 5 (6.9%)  - FSGS: 1 (1.4%) | 2000–2018 | ABO-incompatible: 87 patients | ABO-identical: 114 patients | - Incidence of de novo TMA: 7.5% (15/201)   - ABO-incompatible: 17.2% (15/ 87)  - ABO-identical: 0%   - ABO-incompatibility is a significant risk factor for systemic de novo TMA (odds ratio, 55.9) |
| Wu, 2016  (64) | Germany | Cohort  - Total 62 patients  - AMR- TMA group: 30  - AMR+ TMA group: 32 | - AMR- TMA: 45.1 ± 15.8 - AMR+ TMA: 44.6 ± 17.3 | AMR-TMA: 18/30 (60.0%) AMR+TMA: 24/32 (75.0%) |  | January 2000– December 2012 | AMR-TMA | AMR+ TMA | Graft survival rate   - AMR- TMA   - 1-year post Tx: 82.8% (n = 29)  - 5-year post Tx: 70.4% (n = 27)  - 8-year post Tx: 68.2% (n = 22)   - AMR+ TMA   - 1-year post Tx: 70.0% (n = 32)  - 5-year post Tx: 48.3% (n = 29)  - 8-year post Tx: 28.0% (n = 25) |
| Zarifian,  1999 (65) |  | Kidney and  kidney-pancreas  transplant  recipients  at a  single center,  n = 188 | 34.8 ± 2.1 |  |  | 1994–1996 |  |  |  |

aHUS, atypical hemolytic uremic syndrome; AMR, antibody-mediated rejection; ATG, anti-thymocyte globulin; AZA, azathioprine; CFH, complement factor H; CFI, complement factor I; CMV, cytomegalovirus; CNI, calcineurin inhibitor; CyA, cyclosporine A; DM, diabetes mellitus; ESRD, end-stage renal disease; FK506, tacrolimus; FSGS, focal segmental glomerulosclerosis; HCV, hepatitis C virus; HELLP, hemolysis, elevated liver enzymes, low platelet count; HLA, human leukocyte antigen; HTN; hypertension; HUS, hemolytic uremic syndrome; mTOR, mammalian target of rapamycin; mTORi, mammalian target of rapamycin inhibitor; N/A, not applicable; Okt3, Muromonab-CD3; PCKD, polycystic kidney disease; PEX, plasma exchange; SD, standard deviation; SLE, systemic lupus erythematosus; SRL, sirolimus; TMA, thrombotic microangiopathy; TTP, thrombotic thrombocytopenic purpura; Tx, transplantation; USA, United States of America

**Figure S1. PRISMA flow chart summarizing study identification and selection**

Records identified through database searching (n=229) (PubMed, Embase, Cochrane library)

126 records excluded based on review of titles and abstracts:

Wrong population, outcome, and subject matter: 60

Duplicate: 62

103 full-text articles assessed for eligibility

31 records excluded:

Poster, insufficient data, or editorial protocol

Hand search: 3

75 studies included in qualitative synthesis

Case series or case report: 46

Cohort or single arm study: 29

**Figure S2. Matrix indicating enrolled study type versus published year**


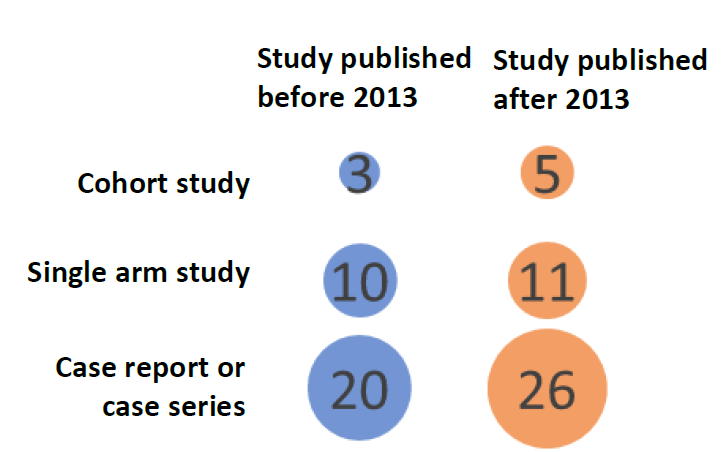


**Figure S3. Matrix indicating immunosuppressants versus demographics, treatments, and outcomes according to case series and reports**


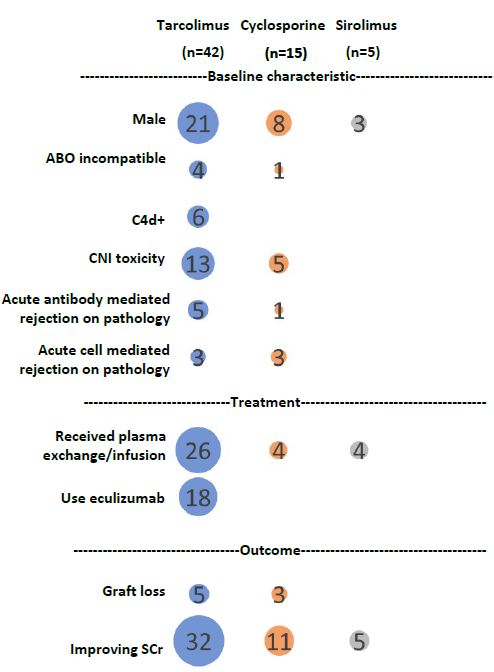


**Figure S4. Incidence of de novo thrombotic microangiopathy within 5 years**


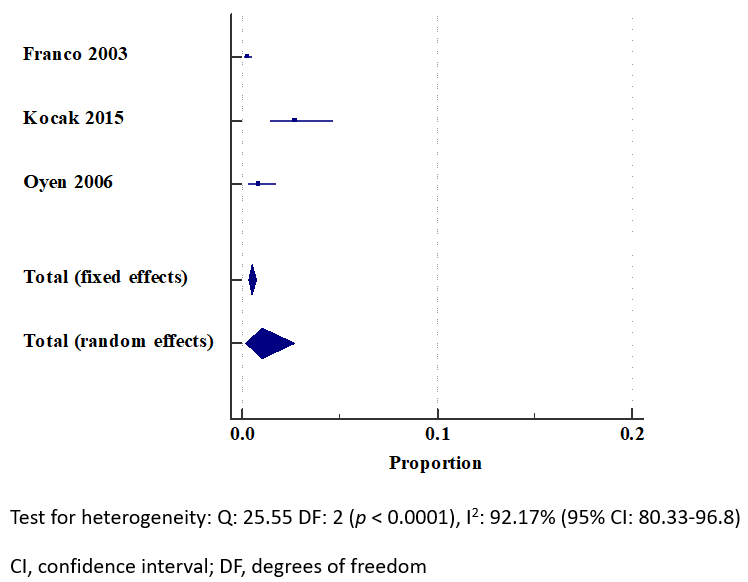


**Figure S5. Incidence of de novo thrombotic microangiopathy between 5 and 10 years**


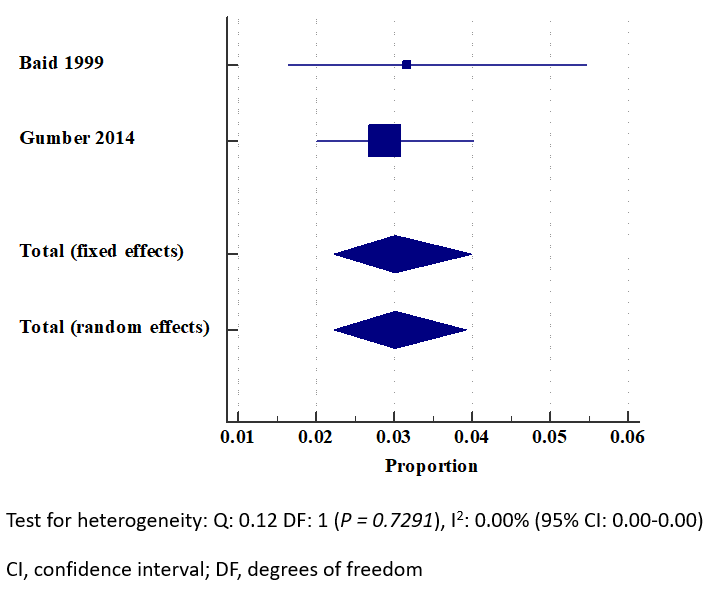


**Figure S6. Incidence of de novo thrombotic microangiopathy more than 10 years**


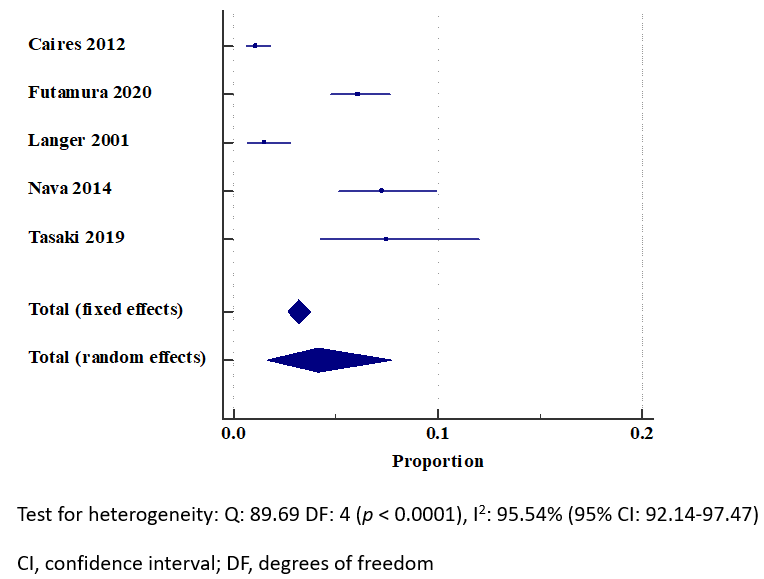


**Figure S7. One year graft survival in patients with de novo thrombotic microangiopathy**


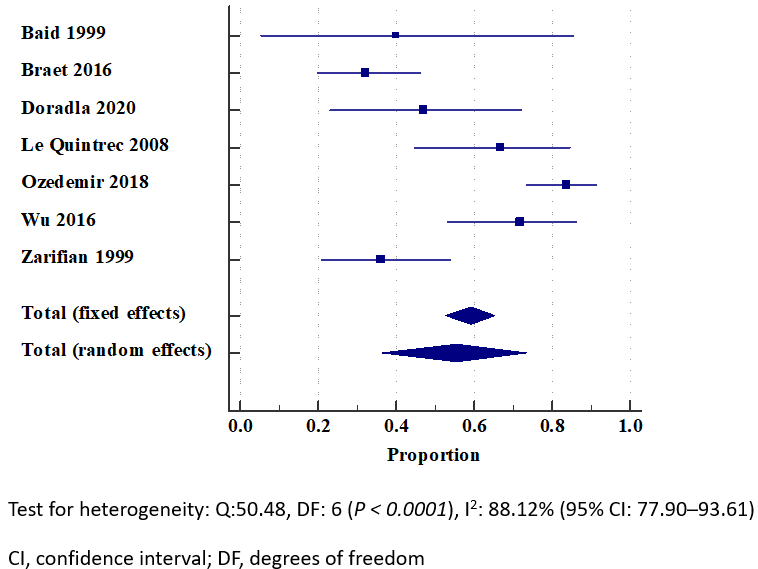

Supplement: Supplementary file 1 [file DataSheet1.doc]
